# Glutamate-dependent ectodomain shedding of neuregulin-1 type II precursors in rat forebrain neurons

**DOI:** 10.1371/journal.pone.0174780

**Published:** 2017-03-28

**Authors:** Yuriko Iwakura, Ran Wang, Naoko Inamura, Kazuaki Araki, Shigeki Higashiyama, Nobuyuki Takei, Hiroyuki Nawa

**Affiliations:** 1 Department of Molecular Neurobiology, Brain Research Institute, Niigata University, Niigata, Japan; 2 Department of Biochemistry and Molecular Genetics, Ehime University, Graduate School of Medicine, Ehime, Japan; University of Rochester School of Medicine and Dentistry, UNITED STATES

## Abstract

The neurotrophic factor neuregulin 1 (NRG1) regulates neuronal development, glial differentiation, and excitatory synapse maturation. NRG1 is synthesized as a membrane-anchored precursor and is then liberated by proteolytic processing or exocytosis. Mature NRG1 then binds to its receptors expressed by neighboring neurons or glial cells. However, the molecular mechanisms that govern this process in the nervous system are not defined in detail. Here we prepared neuron-enriched and glia-enriched cultures from embryonic rat neocortex to investigate the role of neurotransmitters that regulate the liberation/release of NRG1 from the membrane of neurons or glial cells. Using a two-site enzyme immunoassay to detect soluble NRG1, we show that, of various neurotransmitters, glutamate was the most potent inducer of NRG1 release in neuron-enriched cultures. NRG1 release in glia-enriched cultures was relatively limited. Furthermore, among glutamate receptor agonists, N-Methyl-D-Aspartate (NMDA) and kainate (KA), but not AMPA or tACPD, mimicked the effects of glutamate. Similar findings were acquired from analysis of the hippocampus of rats with KA-induced seizures. To evaluate the contribution of members of a disintegrin and metalloproteinase (ADAM) families to NRG1 release, we transfected primary cultures of neurons with cDNA vectors encoding NRG1 types I, II, or III precursors, each tagged with the alkaline phosphatase reporter. Analysis of alkaline phosphatase activity revealed that the NRG1 type II precursor was subjected to tumor necrosis factor-α-converting enzyme (TACE) / a Disintegrin And Metalloproteinase 17 (ADAM17) -dependent ectodomain shedding in a protein kinase C-dependent manner. These results suggest that glutamatergic neurotransmission positively regulates the ectodomain shedding of NRG1 type II precursors and liberates the active NRG1 domain in an activity-dependent manner.

## Introduction

The neurotrophic factor neuregulin 1 (NRG1) is a member of the epidermal growth factor (EGF) family, which is widely distributed along with its receptors (ErbB3, ErbB4) in the central nervous system (CNS) [1–3]. Intense attention has focused on NRG1 since the discovery of its genetic association with schizophrenia [4].

The primary transcript and precursor protein encoded by *NRG1* are expressed by brain neurons and are subject to alternative splicing and proteolytic processing, respectively [5–10]. Evidence indicates that NRG1 isoforms are expressed in neurons and non-neuronal cells in the CNS [11, 12]. NRG1 isoforms include a membrane-anchored form and a soluble form lacking the membrane-spanning region. The membrane-anchored NRG1 precursor is proteolytically processed into the mature soluble form. Although the function of the membrane-anchored form of NRG1 remains to be determined, the soluble isoform of NRG1 stimulates ErbB3 or ErbB4 receptors expressed by neurons and glial cells both *in vitro* and *in vivo* [13–15]. Moreover, the production and release of mature soluble NRG1 is controlled by multiple mechanisms [16]. The last and likely rate-limiting step in the maturation and liberation of NRG1 is proteolytic processing. However, the neural regulators of maturation remain to be characterized.

We investigated the mechanism of shedding and release of the membrane-spanning EGF precursors and heparin-binding EGF-like growth factor (HB-EGF) and found that dopamine as well as glutamate and evoke these events in brain neurons [17–19]. Similarly, the extracellular juxtadomain of membrane-spanning NRG1 precursors is susceptible to proteolytic enzymes such as ADAMs of the matrix metalloproteinase (MMP) family and the β-site amyloid precursor protein cleaving enzyme (BACE) of the aspartic-acid protease family [8–10, 20]. Almost all splice variants of NRG1 precursors retain this juxtamembrane domain and may be shed and released by the above enzymes [14, 21, 22]. However, little information is available on the neural activity-dependent mechanism that regulates ectodomain shedding of individual isoforms of the NRG1 precursors.

In the present study, we investigated how neurotransmission induces the shedding and release of NRG1 in brain cells. For this purpose, we used sensitive ELISA [23, 24] to measure the release of soluble NRG1 from cultured neocortical neurons or glial cells. We transfected neocortical neurons with a vector that expresses NRG1 precursors tagged with a reporter enzyme to identify the neurotransmitters and their receptors that are responsible for the activation of shedding. To estimate which enzyme(s) are involved in shedding, we used inhibitors of the ADAMs [25, 26]. Our findings provide a better understanding of the neurobiological role of glutamatergic neurotransmission in the activation of NRG1 shedding and signaling in the CNS.

## Methods

### Animals

Sprague-Dawley (SD) rats (Japan SLC, Inc., Shizuoka, Japan) were maintained in the animal care facility of Niigata University Brain Research Institute. All rats were housed in acrylic cages (24 x 39 x 19.5 cm) and they had food and water *ad libitum* in a temperature-controlled room (23 ± 2°C) under a 12-h light: 12-h dark cycle (light from 7:00 a.m. to 7:00 p. m.). The Animal Use and Care Committee of Niigata University approved this study and all animal experiments described were carried out in accordance with the institutional guidelines and with those of the National Institutes of Health Guide for the Care and Use of Laboratory Animals (NIH Publications No. 80–23). All efforts were made to minimize discomfort to the rats and the number used.

### Induction of seizures

Kainate (KA; Nacalai Tesque, Kyoto, Japan) was administered to male SD rats (6 weeks old, Japan SLC Inc.). Rats were administered an intraperitoneal injection (i.p.) of KA (20 mg/kg in saline) (KA-treated rats) or injected with saline (control rats). Rats were monitored within 10 min after injection and KA-treated rats exhibited seizures within 30 min after injection. The onset of seizure was characterized according to the Racine’s scale as wet dog shakes, head nodding, and facial clonus [27, 28]. Three hours after KA or saline injection, rats were anesthetized using sodium pentobarbital (50 mg/kg, i.p.) and then decapitated. The rats exhibiting severe seizure such as foaming at the mouth within 20 min after KA injection were promptly euthanized using sodium pentobarbital. Brains were rapidly removed and the hippocampus was quickly isolated and stored at −80°C.

### Cell culture

Cortical tissues were dissected from embryos at embryonic days (E) 18–19 anesthetized using CO_2_, and dissociated using 1 mg/mL papain. To prepare neuron-enriched primary cultures, cells (1.0 × 10^6^ cells/mL) were plated on a dish coated with poly-D-lysine that contained Dulbecco’s modified Eagle medium (DMEM) and 10% fetal bovine serum (FBS). After a 1-h incubation, the medium was replaced with serum-free medium and the cultures were incubated for 7 days as described previously [29]. On day 7 (DIV7), neuron-enriched primary cultures were exposed to 1 μM phorbol 12-myristate 13-acetate (PMA; Merck Millipore, Darmstadt, Germany), 10 μM glutamate (Sigma–Aldrich, St. Louis, MO, USA) 100 μM N-methyl-d-aspartate (NMDA; Nacalai Tesque, Inc.), α-amino-3-hydroxy-5-methyl-4-isoxazolepropionic acid (AMPA; Nacalai Tesque. Inc.), acetylcholine (Nacalai Tesque, Inc.), serotonin (Nacalai Tesque, Inc.), 1-aminocyclopentane-trans-1, 3-dicarboxylic acid (tAPCD, Nacalai Tesque, Inc.), 50 μM L- (+)-2-amino-4-phosphonobutyric acid (L-AP4; Nacalai Tesque, Inc.) or 30 μM dopamine (Sigma–Aldrich) at 37°C. Other cultures were preincubated with 100 nM GM6001 (Merck Millipore), botulinum toxin type A (BoNT/A; a gift from Dr. Kozaki, Osaka Prefecture University), 1 μM calphostin C (Sigma–Aldrich), 10 μM adenosine 3', 5’-cyclicmonophosphothioate (Rp-cAMP; Merck Millipore), 50 μM 2-amino-5-phosphonovalerate (AP5; Tocris Bioscience, Bristol, UK), or 10 μM 6-cyano-7-nitroquinoxaline-2, 3-dione (CNQX; Tocris Bioscience) at 37°C before treatment with glutamate or NMDA.

Glia-enriched cultures were prepared from the same rat cortical tissue. Cells were plated on uncoated dishes containing DMEM and 10% FBS and cultured until reaching 80%–90% confluence. After extensive trypsinization, cells (1.0 × 10^6^ cells/mL) were plated in DMEM containing 10% FBS and incubated for 24 h. After incubation overnight in serum-free media, non-neuronal cell cultures were similarly challenged with glutamate-receptor agonists. Cells were also fixed and processed for immunocytochemistry to detect neuronal and glial markers.

### Enzyme-linked immunoassay

NRG1 levels were measured using an enzyme-linked immunoassay (ELISA) as previously described [17, 23, 24]. Briefly, culture supernatants (i.e., conditioned media) of cortical neurons and non-neuronal cells were similarly centrifuged. Supernatants of conditioned media, as well as the NRG1 standard, were added to the wells of ELISA plates coated with an antigen-capture rabbit anti-NRG1β1 antibody [23]. The captured NRG1 reacted with a guinea pig anti-NRG1β1 antibody [24] and the complexes were detected using a biotinylated anti-guinea pig immunoglobulin (Open Biosystems, Huntsvill, AL, USA) and avidin-β-galactosidase (1:10,000; Rockland Immunochemicals Inc., Gilbertsville, PA, USA). The fluorescence emitted by 4-methylumbelliferyl-β-d-galactoside (Sigma–Aldrich) was measured using a microplate reader (excitation, 490 nm; emission, 530 nm; CORONA Electric, Ibaraki, Japan). The cross-reactivity of the NRG1 immunoassay with other EGF family proteins was <0.1% [23].

### Western blotting analysis

The levels of phospho-ErbB4 and ErbB4 were determined using immunoblot analysis as described previously [17]. Briefly, proteins in cell lysates were reduced and denatured using 5% β-mercaptoethanol and 1% sodium dodecyl sulfate (SDS), separated using SDS polyacrylamide gel electrophoresis and transferred to a polyvinylidene difluoride membrane (Immobilon^TM^; Merck Millipore). The membrane was probed with the anti-phospho-ErbB4 antibody (1:500; Cell Signaling Technology, Danver, MA, USA) and an anti-ErbB4 antibody (1:1,000; Santa Cruz Biotechnology, Inc., Dallas, Texas, USA). After extensive washing, immunocomplexes bound to the membrane were reacted with an anti-rabbit or anti-mouse immunoglobulin conjugated to horseradish peroxidase. Immune complexes were detected using a chemiluminescence reaction (ECL Kit; Amersham Biosciences, Piscataway, NJ, USA). Quantitative densitometric analyses of immunoblots were performed using Image J software (National Institutes of Health, Bethesda, MD, USA).

### Immunocytochemistry

Cortical neuron-enriched cultures and glia-enriched cultures were fixed and processed for immunocytochemistry to detect the expression of neuronal and glial markers using the following antibodies: anti-MAP2 (1:200; Santa Cruz Biotechnology, Inc.), anti-GFAP (1:1,000; DAKO, Glostrup, Denmark), anti-nestin (1:200; Merck Millipore), anti-NeuN (1:200; Merck Millipore), or anti-A2B5 (1:100; Boehringer Mannheim, Ingelheim, Germany). Neuron-enriched cultures transfected with human placental alkaline phosphatase (AP)-tagged NRG1 precursor cDNAs were incubated with an anti-human AP antibody (1:50; DAKO) before fixation. Immunoreactivity was visualized using an Alexa Fluor 546-conjugated anti-mouse or anti-rabbit immunoglobulin, and Alexa Fluor 488-conjugated anti-rabbit immunoglobulin (Vector Laboratories, Burlingame, CA, USA).

### Alkaline phosphatase assay for the analysis of pro-NRG1 shedding

The expression vector containing a cDNA encoding the human NRG1 precursor cDNA tagged with human placental AP was constructed by modifying a cDNA expression vector encoding the HB-EGF precursor [17, 30]. The cDNA fragments encoding each of the three signal sequences and extracellular domains of human NRG1 types I–III were inserted into the multiple cloning site (NotI-XbaI) of the pRc/CMV vector (Invitrogen, Carlsbad, CA, USA). The AP cDNA was ligated to sequences encoding the N-terminus of the EGF and intracellular domains, which were then ligated to the cDNA construct described above comprising the signal sequence and extracellular domain.

Cortical cultures (1 × 10^6^ cells per well) were prepared as described above and grown in 12-well plates. The calcium phosphate transfection method (CalPhos Mammalian Transfection Kit; Clontech Laboratory Inc., Mountain View, CA, USA) modified by Jiang & Chen [31] was used to transfect cortical neurons with the expression vectors carrying the AP-tagged NRG1 precursor cDNAs (pRc-CMV/NRG1-type I-AP, pRc-CMV/NRG1-type II-AP, and pRc-CMV/NRG1-type III-AP). After 48 h, cultured neurons were exposed to PMA or glutamate. In the blocking experiments, cultures were preincubated with AP5, CNQX, or GM6001 before the treatment with PMA or glutamate as described above. The AP activity assay *in vitro* is based on the conversion of colorless *p*-nitrophenyl phosphate (*p*NPP) into yellow *p*-nitrophenol, with a maximum absorption maximum at 405 nm [32]. An aliquot (0.1 mL) of culture supernatant was harvested and heated for 30 min at 60°C to inactivate endogenous AP activity and then mixed with an equal volume of the *p*NPP solution (1 M diethanolamine, 0.01% MgCl_2_, and 1 mg/mL *p*NPP, pH 9.5) and incubated for 30 min at 37°C. The activity of human placental AP (Calbiochem, 410 Unit/mL) was also simultaneously measured with those culture samples as a standard for preparing a calibration curve [17, 30]. Absorbance at 405 nm was measured using a microplate reader (Benchmark, Bio-Rad Laboratories, Hercules, CA, USA).

### Enzyme activity assay for ADAMs

Cortical cultured neurons were homogenized in lysis buffer (20 mM Tris, pH 8.0, 0.06% Brij-35, 0.1% Triton X-100). The lysates were incubated with a fluorescent substrate for tumor necrosis factor-α-converting enzyme (TACE/ADAM17; BIOMOL Research Laboratories Inc., Plymouth Meeting, PA, USA) for 1 h at 37°C in the presence of 20 mM Tris, pH 8.0, and 0.06% Brij-35. The amount of fluorescence was measured using a microplate reader (CORONA Electric) with excitation and emission wavelengths set to 490 nm and 530 nm, respectively.

### Statistical analysis

All values are presented as the mean ± S.D. (standard deviation). The significance of pharmacological effects was evaluated using the Student’s *t*-test (data from two groups) or the Tukey–Kramer significant difference test (multiple groups). *P* values < 0.05 were considered statistically significant.

## Results

### Glutamate and acetylcholine evoke NRG1 release from cultured neocortical neurons

**Fig 1:**
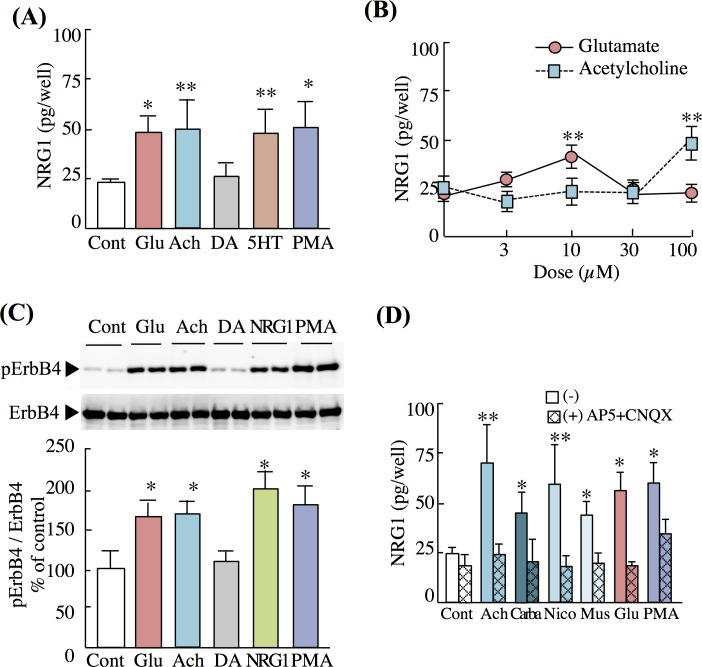
Effects of neurotransmitters on NRG1 release from neuron-enriched rat cortical cultures. (A) Neuron-enriched cultures were treated with control vehicle (Cont), glutamate (Glu, 10 μM, 20 min), acetylcholine (Ach, 100 μM, 20 min), dopamine (DA, 30 μM, 20 min) or serotonin (5HT, 100 μM, 20 min), and PMA (1 μM, 30 min) on day 7 (DIV7). (B) Dose dependency of NRG1 release. Cultures were treated with 0, 10, 30, and 100 μM glutamate or acetylcholine (20 min). (C) Western blotting using anti-phospho-ErbB4 and anti-ErbB4 antibodies. Representative immunoblots are shown. The mean levels of phospho-ErbB4 and ErbB4 immunoreactivities in controls were defined as 100%. (D) Effects of glutamate receptor antagonists and acetylcholine receptor agonists on NRG1 release. Cultures were pretreated with AP5 and CNQX (50 μM and 10 μM, 20 min), then control vehicle (Cont), acetylcholine (Ach, 100 μM, 20 min), carbachole (Carba, 100 μM, 20 min), nicotine (Nico, 100 μM, 20 min), muscarine (Mus, 100 μM, 20 min), glutamate (Glu, 10 μM, 20 min), or PMA (1 μM, 30 min). NRG1 concentrations in culture supernatants were measured using an ELISA. Data represent the mean ± SD (four sister cultures, each); *p < 0.05, **p < 0.01 vs. control vehicle.

We treated cortical neuron-enriched cultures with glutamate, acetylcholine, dopamine, and serotonin and determined their abilities to trigger the shedding or release of NRG1. The shedding/release efficacy of NRG1 was estimated from the amount of soluble and active NRG1 released into the culture medium. Glutamate and acetylcholine induced the release of the highest amounts of soluble NRG1 into the culture media (Fig 1A). The potency of these compounds to liberate NRG1 was comparable with that of the positive-control drug PMA, a protein C kinase (PKC) activator. In contrast, serotonin, but not dopamine, induced the release of NRG1.

The effects of glutamate and acetylcholine on NRG1 release were concentration-dependent and maximum release occurred with 10 μM glutamate or 100 μM acetylcholine (Fig 1B and S1A Fig). In the case of serotonin-evoked NRG1 release, we obtained the maximum response using a 100-μM concentration. However, dopamine at concentrations as high as 100 μM had no effect (S1B Fig). The inability of dopamine to induce NRG1 release contrasts with its ability to induce EGF release from cultured striatal neurons, although both factors belong to the same gene family and share the same protein structure [17]. Further, we found that glutamate, acetylcholine, and serotonin induced the release of similar levels of NRG1 from cultured hippocampal neurons as well (S2 Fig).

Our immunodetection assay did not verify the biological activity of NRG1. Therefore, we analyzed the phosphorylation of the NRG1 receptor ErbB4 in the same cultures. We found that exposure to glutamate and acetylcholine increased the level of ErbB4 phosphorylation, which was consistent with that triggered by authentic NRG1 (Fig 1C). These results indicate that these neurotransmitters induce the release of biologically active NRG1.

Acetylcholine receptors are expressed at excitatory synaptic terminals and regulate the presynaptic release of glutamate or postsynaptic NMDA responses [33–36]. Using the most effective dose of acetylcholine, we assumed that acetylcholine-triggered NRG1 release might be mediated by acetylcholine, which secondarily evokes glutamate release. To test this possibility, we pretreated cortical cultures with antagonists of ionotropic glutamate receptors (CNQX and AP5) and then challenged them with acetylcholine and the acetylcholine derivatives calbacol, muscarine, and nicotine. Pretreatment with these antagonists not only blocked the effects of glutamate but also abolished acetylcholine-dependent NRG1 release (Fig 1D). The same evidence was obtained in cultured hippocampal neurons (S2 Fig).

### NMDA is a potent regulator of NRG1 release

**Fig 2:**
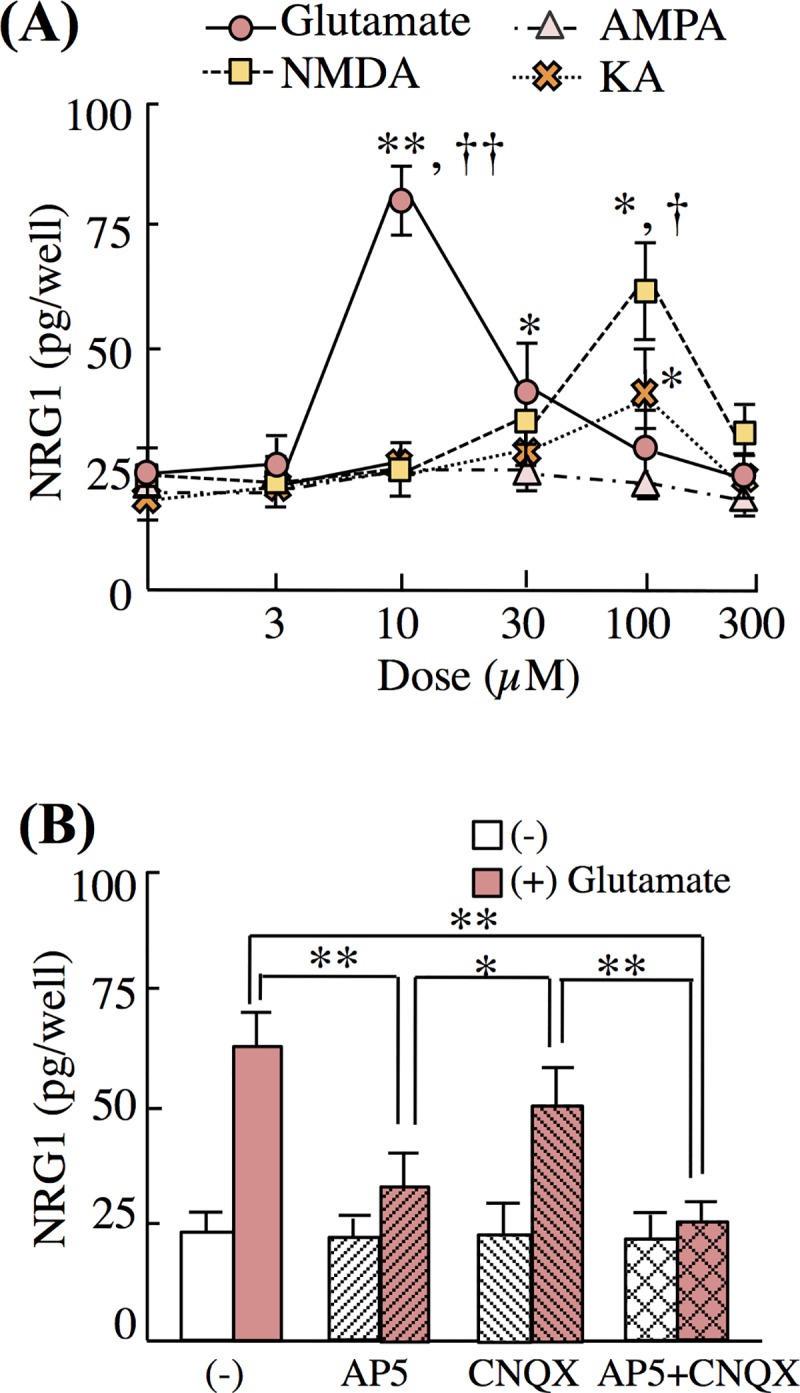
Effects of glutamate receptor agonists and antagonists on NRG1 release. (A) Neuron-enriched cultures were treated with 0, 3, 10, 30, 100, and 300 μM of glutamate, NMDA, AMPA, or KA for 20 min. Data represent the mean ± SD; *p < 0.05, **p < 0.01 vs. control vehicle; ^†^*p* < 0.05, ^††^*p* < 0.01 vs. 100 μM of KA. (B) Neuron-enriched cultures were pretreated with AP5 (50 μM, 20 min), CNQX (10 μM, 20 min), or both prior to challenge with control vehicle (Cont) or glutamate (Glu, 10 μM) for 20 min. Concentrations of NRG1 released to culture supernatants were measured using ELISA (four sister cultures each). Data represent the mean ± SD; *p < 0.05, **p < 0.01.

To further characterize glutamate-dependent NRG1 release, we determined which glutamate receptor subtypes mediate NRG1 release, employing their specific receptor agonists. We challenged neuron-enriched cultures with various doses of NMDA, KA, AMPA, or glutamate and similarly determined the amounts of NRG1 release. The maximum levels of NRG1 release were achieved using 10 μM glutamate, 100 μM NMDA, and 100 μM KA. The effect of 100 μM NMDA was statistically as potent as 10 μM glutamate whereas 100 μM KA was inferior to both NMDA and glutamate (Fig 2A). To validate these results, we tested the attenuation of the evoked NRG1 release by ionotropic glutamate receptor antagonists. AP5, which is a selective GluN antagonist, inhibited the effects of glutamate similarly to CNQX plus AP5. However, CNQX, which is a non-NMDA glutamate receptor antagonist, did not suppress the effect of glutamate (Fig 2B). These results suggest that NMDA, as well as glutamate, is a potent regulator of NRG1 release.

### The metalloproteinase inhibitor attenuates NRG1 release

**Fig 3:**
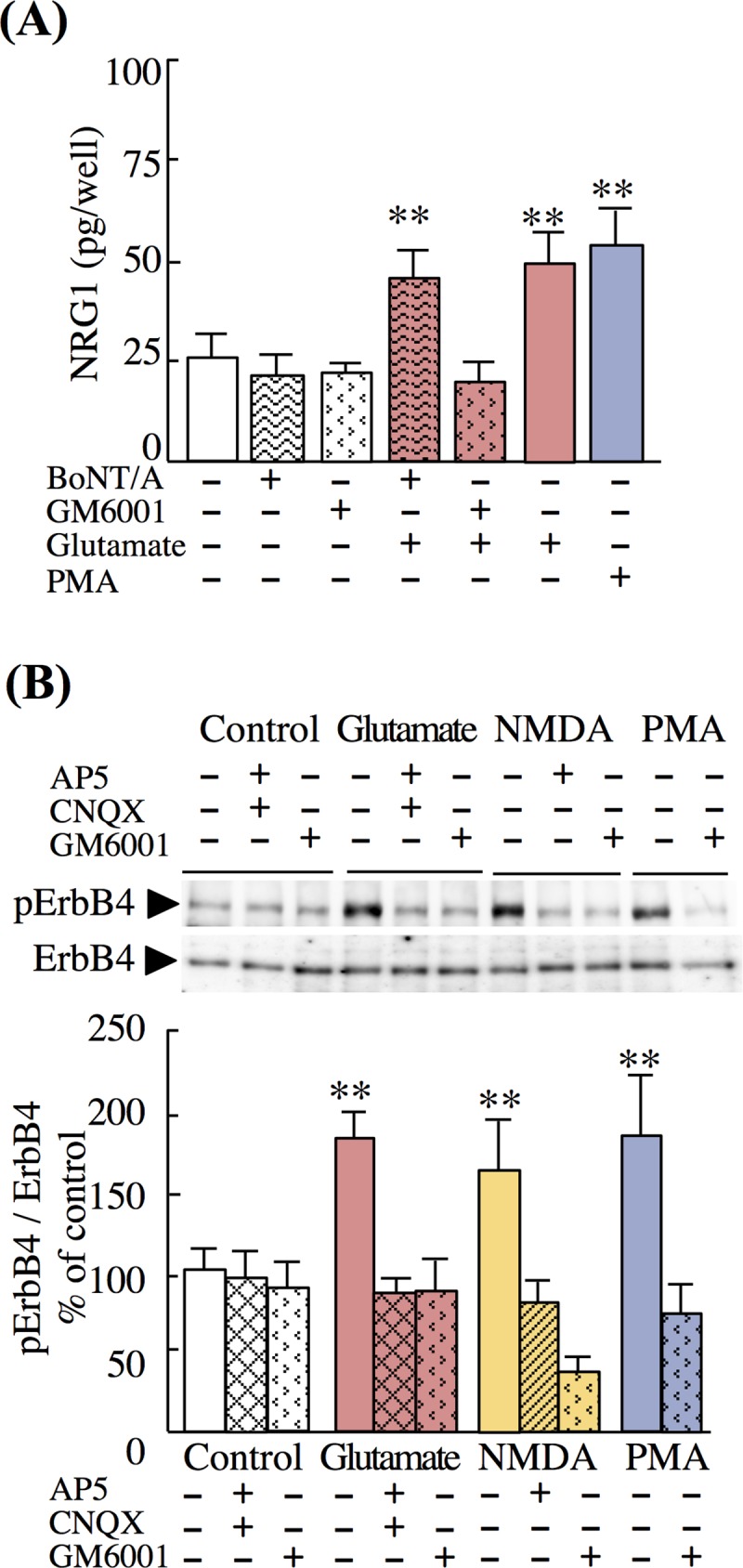
Effects of botulinum toxin and an MMP inhibitor on NRG1 release. (A) Neuron-enriched cultures were pretreated with GM6001 (100 nM, 1 h), BoNT/A (100 nM, 3 h), or control vehicle prior to challenge with vehicle, glutamate (10 μM, 20 min), or PMA (1 μM, 30 min). NRG1 concentrations in culture supernatants were measured using ELISA. (B) Neuron-enriched cultures were pretreated with GM6001, AP5, or CNQX and then challenged with control vehicle, glutamate, NMDA, or PMA as described above. Cell lysates were subjected to Western blotting using anti-phospho-ErbB4 and anti-ErbB4 antibodies. Representative immunoblots are shown. Data represent the mean ± SD (four sister cultures each); **p < 0.01 vs. control vehicle.

Almost all neurotrophic factors are released from neuronal cells to the extracellular space through conventional vesicular membrane fusion or extracellular-domain shedding of their membrane-spanning precursors [8–10]. We employed an inhibitor of vesicular fusion, BoNT/A, and the broad MMP inhibitor GM6001 [37] to independently inhibit vesicular release and ectodomain shedding, respectively (Fig 3). Pretreatment with either GM6001 or BoNT/A alone had no significant effect on basal NRG1 release. However, pretreatment with GM6001 blocked glutamate- or NMDA-induced NRG1 release into the medium, whereas BoNT/A did not (Fig 3A). We validated these effects by simultaneously measuring the phosphorylation of ErbB4. GM6001 attenuated ErbB4 phosphorylation induced by glutamate, NMDA, and PMA, as well as by AP5, CNQX, and both AP5 and CNQX (Fig 3B).

β-secretase (β-site amyloid precursor protein cleaving enzyme 1, BACE1) is a member of the pepsin-like family of aspartic proteinases. BACE1 is generally expressed in intracellular vesicles and cleaves NRG1 [21, 38, 39]. Therefore, we employed a selective BACE inhibitor (BACE inhibitor II) and a BACE substrate analogue (BACE inhibitor III). Pretreatment with either BACE inhibitor II or inhibitor III did not inhibit glutamate- or NMDA-dependent NRG1 release (S3 Fig). These results suggest that glutamate-induced NRG1 release involves the activation of MMP-like enzyme(s) and mainly depends on ectodomain shedding.

### NRG1 is released from neurons rather than from glial cells

To determine the phenotypes of cultured cortical cells, we performed immunohistochemical analyses of markers of neurons (MAP2, NeuN), astrocytes (GFAP), neural precursors (nestin), and glial progenitors (A2B5) (Table 1 and S4B–S4I Fig). Although >70% of cells in the neuron-enriched culture expressed MAP2 and NeuN, there were significant populations of nestin-positive or GFAP-positive cells. Therefore, we also evaluated the contribution of NRG1 release from glial cells, which are known to express glutamate receptors [40–42], as well as NRG1 [11, 43]. For this purpose, we prepared glial cell-enriched cultures from the same tissue source. These cultures comprised GFAP-positive astrocytes (88.6 ± 6.8%), nestin-positive neural precursors (72.8% ± 7.8%), and A2B5-positive glial precursors (16.4% ± 6.4%; Table 1). We added glutamate and glutamate receptor antagonists to these cultures and measured NRG1 released into the culture media. There were no significant effects of the glutamate analogues, except KA, on NRG1 release (S4A Fig). However, the absolute concentrations of NRG1 released by KA were lower compared with those released from neuron-enriched cell cultures (S4A Fig). These findings suggest that NRG1 is released exclusively from cultured neurons present in neocortical cultures.

**Table 1:** Cell populations in cortical neuron- or glia-enriched cultures.

% of total cell number	MAP2	GFAP	Nestin	NeuN	A2B5
Neuron-enriched culture	94.8 ± 2.1	3.7 ± 1.4	19.9 ± 4.7	73.6 ± 7.7	ND
Glia-enriched culture	5.9 ± 2.9	88.6 ± 6.8	72.8 ± 7.8	ND	16.4 ± 6.4

Cells in neuron-enriched cultures (Fig 1) and in glia-enriched cultures (Fig 2) were immunostained with anti-MAP2, anti-GFAP, anti-nestin, anti-NeuN (for neuron-enriched cultures), or anti-A2B5 (for non-neuronal cell-enriched cultures) antibodies. Data represent the mean ± SD (four sister cultures). ND, no data. Representative images are shown in S3 Fig.

#### NMDA and KA evoke ectodomain shedding of NRG1 type II precursors

**Fig 4:**
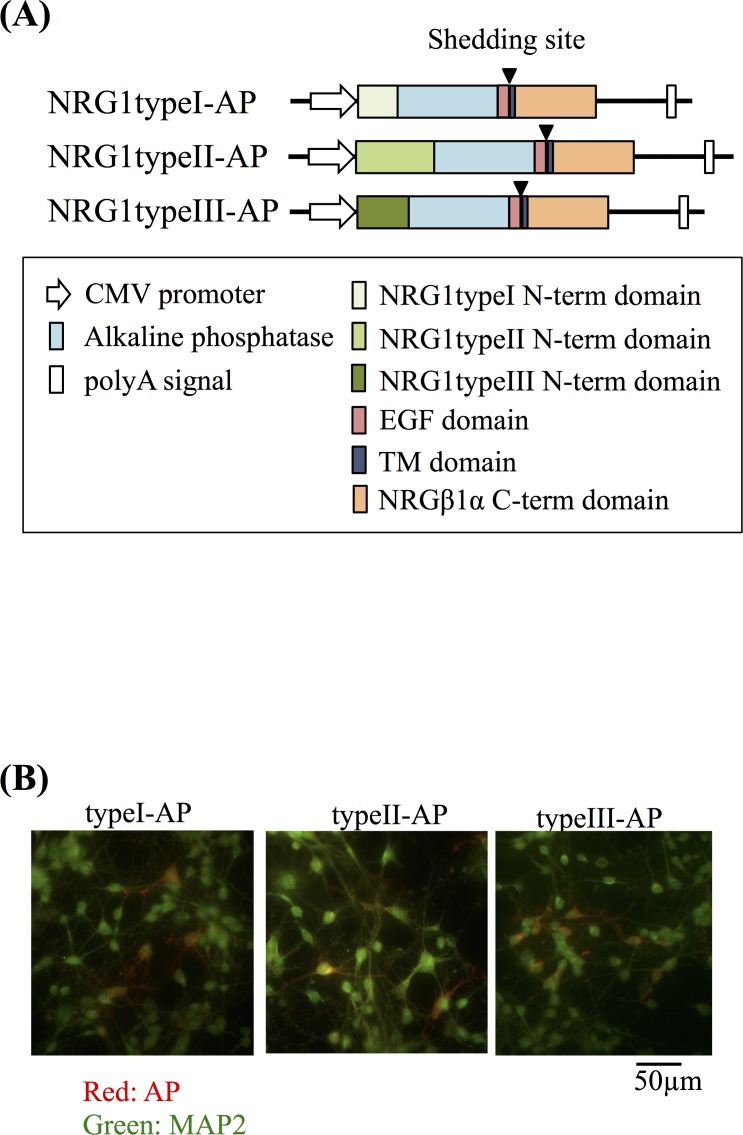
Gene structures of expression vectors encoding AP-tagged types I–III NRG1 precursors. (A) Diagram of the pRc-CMV/NRG1-AP expression vectors. The sequences encoding the human placental AP tag (blue), NRG1 type I–III-specific N-terminal domains (orange, type I; red, type II; yellow, type III). The EGF (light green) and transmembrane domains (deep green) are marked. (B) Following transfection of cells with individual expression vectors, AP immunoreactivity on the surface of living cells was visualized together with MAP2. Live cortical neurons expressing AP-tagged NRG1 type I-III precursors were treated with the anti-AP antibody to visualize NRG1 precursors on the cell surface. After fixation, cells were incubated with a MAP2 antibody followed by fluorescent secondary antibodies (red for AP and green for MAP2). Scale bar = 50 μm.

**Fig 5:**
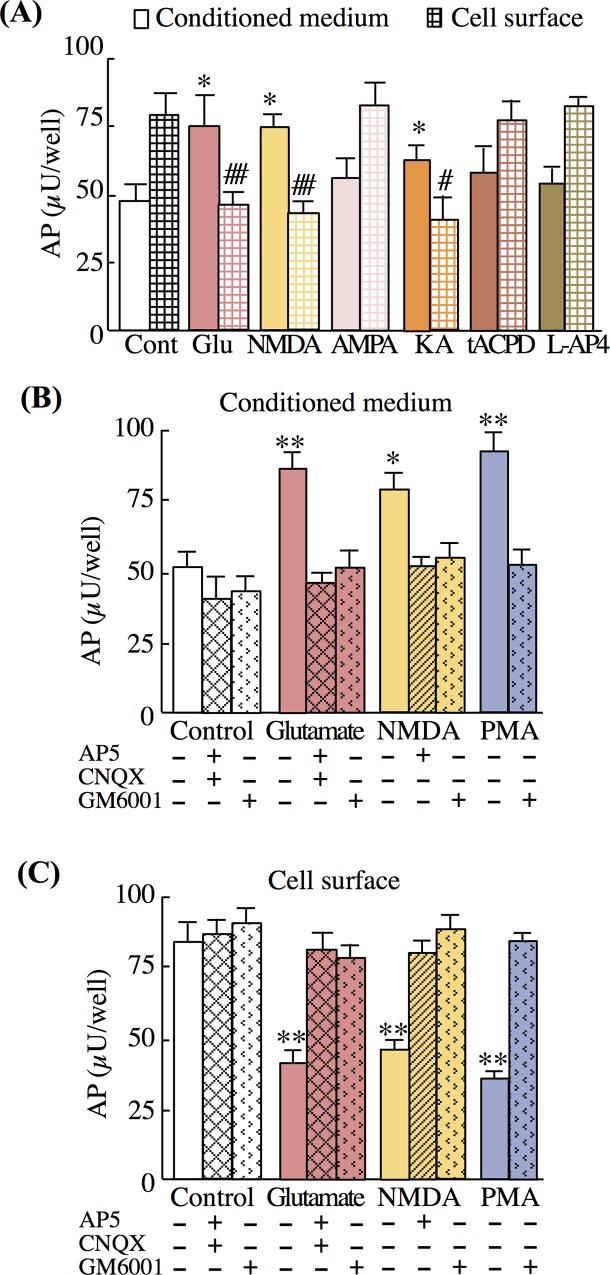
Glutamate receptor agonists increase type II NRG1 release. (A) After the transfection of cells with the NRG1 type II-AP expression vector, neuron-enriched cultures were treated with control vehicle (saline), glutamate (Glu, 10 μM), NMDA (100 μM), AMPA (100 μM), KA (100 μM), tAPCD (100 μM), or L-AP4 (50 μM) for 20 min as described above. The enzyme activities of the AP tag in conditioned medium and at the cell surface are displayed. (B, C) Effects of the glutamate receptor antagonists (AP5, CNQX) on AP activity in conditioned medium (B) and on the cell surface (C) are shown. Data represent the mean ± SD (four sister cultures each); *p < 0.05, **p < 0.01 vs. control vehicle.

Alternative splicing of *NRG1* produces six distinct precursor protein isoforms [11, 13, 44]. Among those, NRG1 type I–III precursors are abundant in the CNS [11]. To further explore the molecular mechanism underlying glutamate-dependent NRG1 release, we transfected cultured neurons with vectors that express NRG1 type I–III tagged with AP [17] (Fig 4A). Immunostaining of live cells with anti-AP antibodies revealed that NRG1 type I–III precursors were expressed on the surface of 5.2% ± 3.3%, 4.6% ± 4.4%, and 5.8% ± 4.1% of cultured neurons, respectively (Fig 4B). However, neurons transfected with the expression vector encoding NRG1 type I and III, but not NRG1 type II, failed to exhibit significant responses to glutamate exposure. The changes in AP expression on the cell surface were reproduced using live-cell immunostaining with the anti-AP antibody (S5 Fig). There were significant decreases in AP-like immunoreactivity on the cell surface of cells transfected with the type II-AP expression vector (S5A Fig). Therefore, we focused on the NRG1 type II to further characterize the neurotransmitter regulation of its ectodomain shedding/release (Fig 5). Treatment with glutamate, NMDA, or KA, but not with AMPA, tAPCD, or L-AP4, similarly increased AP activity in culture supernatants (Fig 5A). Conversely, these glutamate analogues reduced the AP activity retained at the cell surface. The effects of glutamate were attenuated by glutamate-receptor blockers and the MMP inhibitor (Fig 5B and 5C). These results indicate that the excitatory neurotransmitter glutamate is a potent regulator of the ectodomain shedding and release of NRG1 type II precursors.

#### Ectodomain shedding of NRG1 type II precursors requires protein kinase C activation followed by ADAM17 or ADAM9 activity

**Fig 6:**
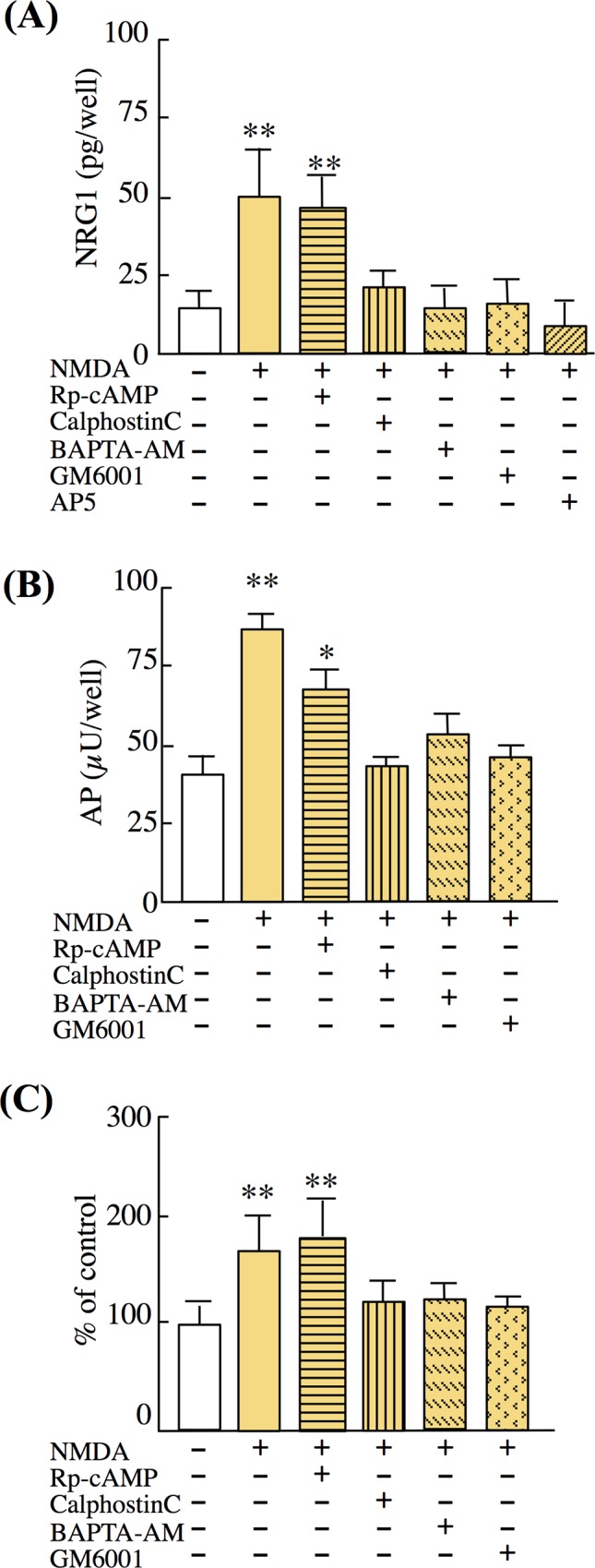
Contribution of protein kinase A/C to NMDA-triggered NRG1 release. On DIV7, neuron-enriched cultures were pretreated with calphostin C (calC, 1 μM, 30 min), Rp-cAMP (10 μM, 30 min), GM6001 (100 nM, 1 h), or AP5 (50 μM, 20 min) and then challenged with control vehicle or NMDA (100 μM, 20 min). (A) NRG1 concentrations in culture supernatants were measured using ELISA. (B) The enzyme activities of the AP tag in culture supernatants were measured. (C) ADAM activity in cultured cells was measured (see Materials and Methods). Data represent the mean ± SD (four sister cultures each); *p < 0.05, **p < 0.01 vs. control vehicle.

Membrane-anchored MMPs require calcium signaling and subsequent protein kinase activation [45, 46]. To assess the contribution of PKC and PKA to shedding, we employed their activator (PMA) and inhibitors (calphostin C and Rp-cAMP) [47, 48] (Fig 6). Pretreatment of neuronal cultures with calphostin C attenuated the NMDA-induced total NRG1 protein release (Fig 6A). In contrast, the PKA inhibitor Rp-cAMP had no significant effect. Pretreatment of cultures with calphostin C similarly decreased the release of the AP tag attached to NRG1 type II (Fig 6B). The same drug dependency was observed in the ADAM17 activity assay; NMDA challenge increased ADAM17 activity, which was reversed by GM6001. In contrast, Rp-cAMP failed to affect ADAM activity (Fig 6C).

However, the molecular specificity of the above inhibitor GM6001 and the ADAM activity assay substrate are controversial [49–52]. Therefore, we employed TAPI-0, a more specific inhibitor of ADAM17 and ADAM9. Pretreatment with TAPI-0 inhibited NMDA-dependent ectodomain shedding of NRG1 type II precursors as monitored with AP activity (S6 Fig). These results suggest that ADAM17 or ADAM9, at least, contribute to the NMDA-dependent ectodomain shedding of NRG1 type II precursors.

#### Kainate-induced seizures cause ectodomain shedding/release of NRG1

**Fig 7:**
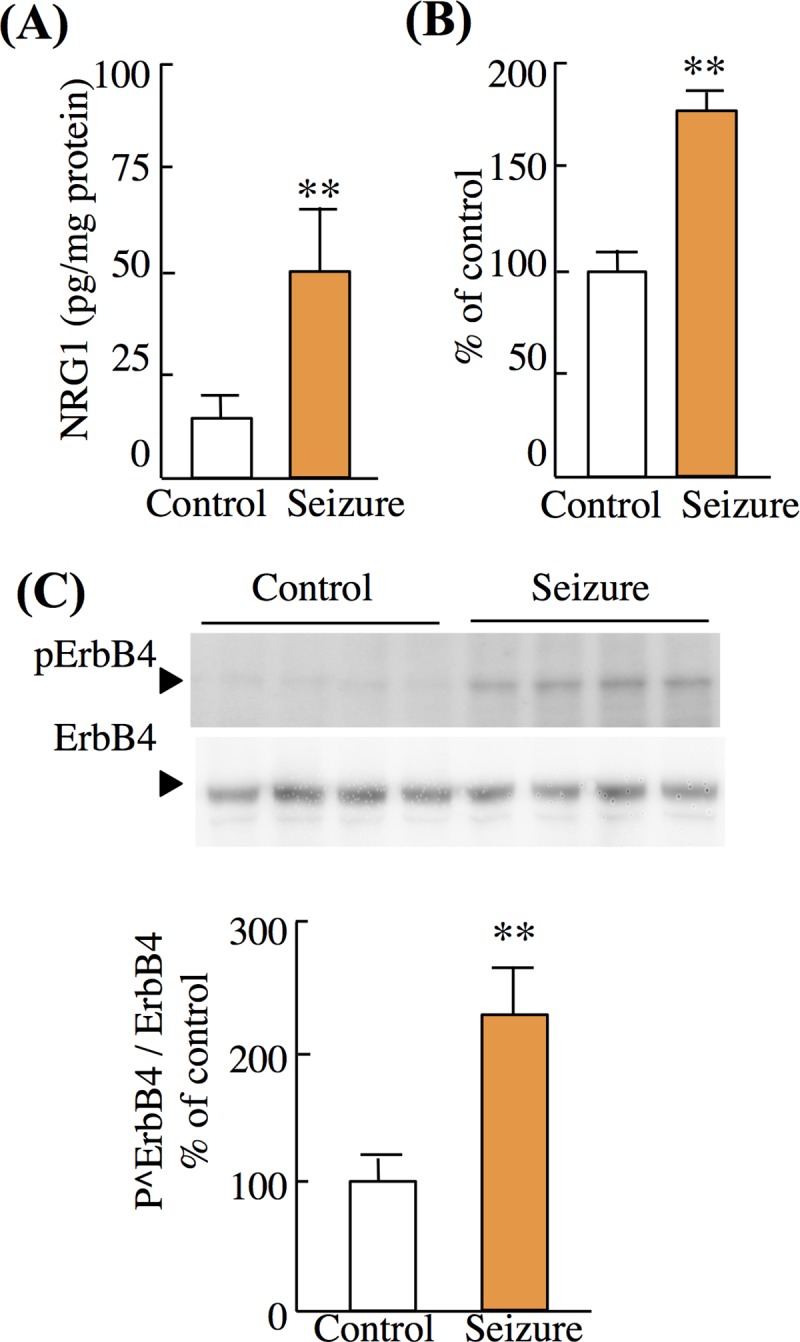
NRG1 release and ErbB4 phosphorylation following KA receptor activation *in vivo*. (A) Levels of soluble-free NRG1 in the hippocampus were determined in epileptic and control rats. (B) Total ADAM activity in hippocampal tissue lysates as described in Fig 5. (C) Crude hippocampal membranes were subjected to Western blotting using anti-phospho-ErbB4 and anti-ErbB4 antibodies. The mean levels of phospho-ErbB4 and ErbB4 immunoreactivities in controls were defined as 100%. Data represent mean ± SD (four rats per group); *p < 0.05, **p < 0.01, vs. saline-treated control group.

The preceding results from culture experiments might only reflect the responses of immature neurons *in vitro*. To exclude this possibility, we investigated the levels of soluble NRG1, ErbB4 phosphorylation, and ADAM17 activity in the hippocampus of adult rats. Tissue lysates were prepared from control and epileptic rats, and these indices were compared to characterize the activity-dependent regulation of NRG1 ectodomain shedding. The seizure-exposed hippocampus showed significant increases in the levels of soluble NRG1, as well those of phosphorylated ErbB4 (Fig 7A and 7C). In parallel, there was a significant increase in ADAM17/ADAM9 activity in the seizure group (Fig 7B). These results suggest that excitatory neurotransmission presumably regulates *in vivo* NRG1 release/shedding in an ADAM-dependent fashion.

## Discussion

**Fig 8:**
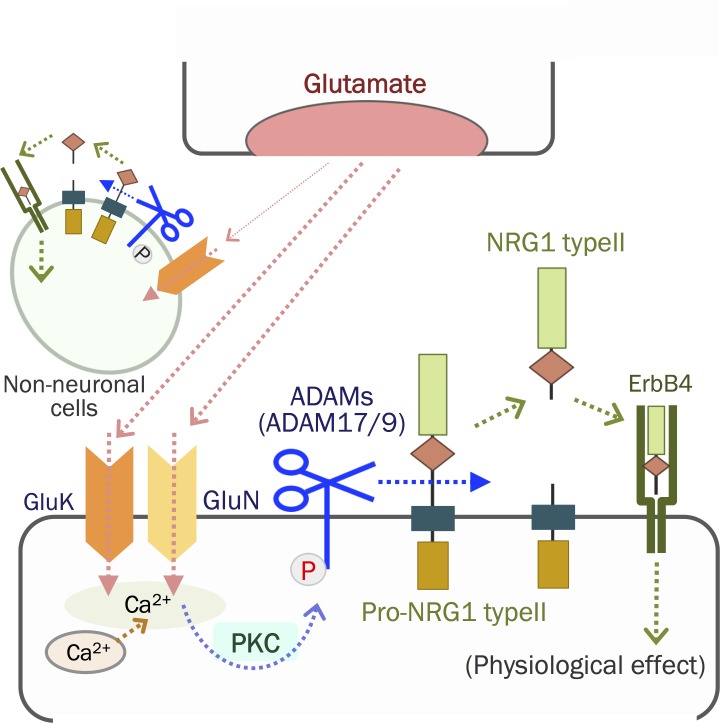
Neuron–target interactions mediated by glutamate and the NRG1 type II isoform. Glutamate released from presynaptic terminals binds to postsynaptic glutamate receptors and activates ADAMs in neurons (e.g., ADAM17) via PKC. ADAMs then cleave the ectodomain of the NRG1 type II precursor and liberates mature NRG1 type II. Free NRG1 type II binds ErbB4 receptors in neurons or neighboring neurons, presumably leading to glutamatergic development, maturation, or both.

In the present study, we explored how neurotransmitters evoke and regulate NRG1 release, primarily aimed at validating the role of its ectodomain shedding. Our significant findings are as follows: (1) Glutamate is a potent inducer of NRG1 release from cultured neocortical and hippocampal neurons; (2) NRG1 release from cultured glial cells is limited; (3) the activation of the NMDA receptor, kainite receptors, or both is involved in NRG1 release; (4) NRG1 release is inhibited by an MPP inhibitor but not by an exocytosis inhibitor; (5) among the membrane-anchored NRG1 precursors, we were able to confirm that the type II precursor is subjected to glutamate-induced ectodomain shedding; (6) and the shedding of the NRG1 type II precursor requires activation of the NMDA receptor followed by PKC activation and ADAM17/ADAM9 enzyme activity. Consistent with these findings, we detected significant increases in soluble NRG1 and ErbB4 phosphorylation in rats with epileptic seizures. These findings suggest that excitatory neurotransmission is a major regulator of NRG1 ectodomain shedding and release in the brain (Fig 8).

### Glutamate-dependent activation of ADAMs

The activation of ADAMs requires PKC signaling triggered by calcium influx [53, 54] and results in the ectodomain shedding of NRG1 precursors in peripheral organs [26, 46, 55, 56]. In agreement with these reports, NMDA potently upregulated the ectodomain shedding of the NRG1 type II precursor in cultured neurons, which activated NMDA glutamate receptor channels and potentiated Ca^2+^-calmodulin and PKC [57].

We did not detect significant increases in NRG1 release from neuron-enriched cultures treated with AMPA and tACPD, which is discordant with previous reports on ADAM activation. For example, Hoey et al. found that the activation of AMPA receptor channels induces ADAM potentiation followed by ectodomain shedding of the amyloid precursor protein [58]. Cho et al. found that the activation of mGluR receptors potentiates ADAM17 and triggers ectodomain shedding of the neuronal pentraxin receptor [59]. These reports demonstrate that AMPA receptors, as well as metabotropic glutamate receptors, can activate ADAMs, suggesting the potential of NRG1 shedding. Why their ligands AMPA and tACPD failed to induce ectodomain shedding of NRG1 in the present study remains to be illustrated.

### Neurotransmitters induce NRG1 release and shedding

Among the members of the EGF family, the membrane anchored HB-EGF precursor has been most extensively investigated. Ectodomain shedding is the rate-limiting step in the release of HB-EGF and its transactivation of EGF receptor. The activation of GPCRs, in particular G_q/I_, evokes EGF receptor transactivation, rerouting ADAM17 and ADAM15 [60]. In contrast to the peripheral mechanism for HB-EGF shedding, in the present study, ectodomain shedding of NRG1 precursors from brain neurons was not induced by GPCR ligands such as dopamine and serotonin. These findings are consistent with those published previously by our group that show that excitatory neurotransmission via GluN and GluK is the major regulator of ectodomain shedding of the HB-EGF precursor in brain neurons [30]. We also detect the NRG1 shedding triggered by GluN and GluK activation in the rat cortical culture, but NMDA produce NRG1 shedding more than KA dose. However, the up-regulation of NRG1 shedding is also detected in the KA-induced seizure rat. Can the discordance between studies be explained by the difference between the peripheral and central nervous systems? This is unlikely because the membrane-anchored EGF precursor is subject to shedding in a dopamine-dependent manner in cultured neurons [17]. In this experimental system, however, the most marked shedding of the EGF precursor is observed in striatal neurons, which may represent the response of inhibitory GABA neurons [61, 62]. Accordingly, we assume that the neurotransmitter species that induce ectodomain shedding may differ among neuronal populations in the CNS, depending upon their expression of GPCR receptor subtypes.

NRG1 is implicated in excitatory synaptic plasticity via ErbB4 activation [63–67]. In our experiments, activation of GluN and GluK accelerates the shedding of NRG1 type II. Vullhorst et al. reported that NMDA triggers NRG2 shedding, creating a negative feedback loop between NRG2/ErbB4 and NMDA/GluN signaling [68]. A similar interaction may exist between NRG1/ErbB4 and NMDA/GluN via NRG1 type II shedding in developing cortical neurons. NRG1 type II hypomorphic rats and GluN2 deletion mice exhibit impairment in anxiety-like behavior and cognitive function [69–72]. This shedding mechanism of NRG1 type II may contribute to such physiological function in developing cortical neurons.

### Subcellular localization of NRG1 shedding and processing

Membrane-anchored NRG1 precursors can be cleaved by proteases such as MMPs, ADAMs, nardilysin, BACE, and neuropsin [9, 39, 56, 73, 74]. In cultured cortical neurons, ADAM17/ADAM9 can cleave the ectodomain of the NRG1 type II precursors to liberate the ectodomain, which is the main process described here. The concomitant decrease in cell-surface activity of the AP tag suggests that ectodomain shedding occurs at the cell surface. Similarly, the exocytosis blocker BoNT/A, excluding a contribution of the vesicular release of NRG1, did not attenuate this NRG1 type II release. However, the distinct types of enzymes responsible for this shedding might illustrate the reported differences in the subcellular localization of NRG1 shedding and processing [14, 26, 39, 56]. Ectodomain shedding of NRG1 typeI occurs in Golgi bodies by ADAM19, and in endosome by BACE1 and a clathrin-binding protein calcyon [21, 55]. The metalloproteinase nardilysin interacts with BACE1 and contribute to NRG1 shedding in endocytic pathways [73, 75, 76]. BACE inhibitors did not suppressed NMDA-dependent NRG1 release in developing cortical neuron in contrast with TACE inhibitors. Nardilysin also forms a complex with TACE and mediates NRG1 shedding on the cell surface[73, 75, 76]. TACE-mediated ectodomain shedding of NRG1 might be occurred mainly on the cell surface.

Considering that this NRG1 type II shedding is facilitated by GluN, we assume that this NRG1 type II shedding mediated by glutamate might be occurring at the postsynapse (Fig 8). However, GluNs also express in the presynaptic terminal of GABAergic interneuron in the retinotectal system and Cerebellar cortex[77, 78]. Presynaptic NRG1 type I or type II precursor interacts with postsynaptic ErbB4 in a trans-synaptic manner [79]. NRG1 shedding mediated by synaptic plasticity may use multiple mechanisms attributed to the heterogeneity and distribution of NRG1 isoforms, shedding proteases and neurotransmitter receptors.

### Limited glial contribution to glutamate-induced NRG1 release

In the present study, we found that cultures of mixed glia released limited amounts of NRG1. Therefore, we conclude that neurons in the cultures were the likely source of ectodomain shedding required for NRG1 release. However, the expression of NRG1 is not limited to neurons. Significant levels of *NRG1* mRNAs are detected in glial cell populations [11]. Among the isoforms of NRG1 precursors generated through alternative splicing, type I NRG1 precursors are highly expressed in glial cells, and types II and III precursors are present in neuronal populations [11, 43]. These findings are consistent with our demonstration that only the NRG1 type II precursor was subject to glutamate-dependent shedding and release in our neuron-enriched cultures. However, we detected KA-induced release of limited amounts of NRG1 from mixed glial cells. The aspartate protease BACE is expressed by glial cells, which does not exclude the possibility that the ectodomain shedding of NRG1 may recruite serine- or aspartate-proteases as well [80, 81]. Therefore, we speculate that the non-neuronal cell population employs a distinct mechanism of NRG1 precursor processing and release [11, 82–84].

## Conclusion

The present study indicates that the excitatory neurotransmitter glutamate is a potent regulator of NRG1 release and shedding from developing neocortical and hippocampal neurons. NRG1 release is partly attributable to the ectodomain shedding of NRG1 type II precursors that is triggered by NMDA or KA glutamate receptors. The shedding process is likely to involve the activation of PKC and ADAM17/ADAM9. Accordingly, glutamate-driven ectodomain shedding of NRG1 could contribute to activity-dependent neural plasticity or development.

## Supporting information

S1 Fig Serotonin increases NRG1 release in a dose-dependent manner in cortical neuron-enriched cultures. (A) Cultures were treated with 0, 30, 100, 300, 1,000 μM of acetylcholine for 20 min at 37°C. NRG1 concentrations in culture supernatants were measured using ELISA. (B) Cultures were treated with 0, 10, 30, and 100 μM of dopamine or serotonin (5HT). NRG1 concentrations in culture supernatants were measured using ELISA. Data represent the mean ± SD (four sister cultures each); *p < 0.05, **p < 0.01 vs. control vehicle.
(TIFF)

S2 Fig Glutamate and acetylcholine increase NRG1 release in hippocampal neuron-enriched cultures. (A) Cultures were treated with control vehicle (Cont), PMA, glutamate (Glu), acetylcholine (Ach), dopamine (DA), or serotonin (5HT) on DIV7. (B) Cultures were treated with 0, 10, 30, and 100 μM of glutamate or acetylcholine. (C) Effects of glutamate receptor antagonists and acetylcholine receptor agonists on NRG1 release. On DIV7, cultures were pretreated with AP5 and CNQX or left untreated, and then challenged with control vehicle (Cont), acetylcholine (Ach), carbachole (Carba), nicotine (Nico), glutamate (Glu), or PMA. NRG1 concentrations in culture supernatants were measured using ELISA. Data represent the mean ± SD (four sister cultures each); *p < 0.05, **p < 0.01 vs. control vehicle.
(TIFF)

S3 Fig BACE inhibitor II and III have no effect on NRG1 release in cortical neuron-enriched cultures. (A) On DIV7, cultures were pretreated with BACE inhibitor II (50 μM, 2 h) and then challenged with control vehicle (Cont), glutamate (10 μM, 20 min), or NMDA (100 μM, 20 min). NRG1 concentrations in culture supernatants were measured using ELISA. (B) On DIV7, cultures were pretreated with BACE inhibitor III (10 μM, 2 h) and then challenged with control vehicle (Control), glutamate (10 μM, 20 min), or NMDA (100 μM, 20 min). NRG1 concentrations in culture supernatants were measured using ELISA. Data represent the mean ± SD (four sister cultures each); *p < 0.05, **p < 0.01 vs. control vehicle.
(TIFF)

S4 Fig Glutamate and NMDA have no effect on NRG1 release in cortical glia-enriched cultures. **(A)** Glia-enriched cultures were challenged with control vehicle (Cont), glutamate (Glu, 10 μM, 20 min), NMDA, AMPA, KA (100 μM, 20 min), or PMA (1 μM, 30 min). NRG1 concentrations in culture supernatants were measured using ELISA. Data represent the mean ± SD (four sister cultures each); *p < 0.05, **p < 0.01 vs. control vehicle. (B–I) Characterization of neuron-enriched and glia-enriched cultures prepared from embryonic cortical tissues. Neuron-enriched cultures were immunostained with the following antibodies: anti-MAP2 (B), anti-GFAP (C), anti-nestin (D), and anti-NeuN (E), followed by anti-mouse or anti-rabbit immunoglobulin fluorescent secondary antibodies. Glia-enriched cultures were immunostained with the following antibodies: anti-MAP2 (F), anti-GFAP (G), anti-nestin (H), and anti-A2B5 (I), followed by anti-mouse or anti-rabbit immunoglobulin fluorescent secondary antibodies. The frequency of individual cell types is shown in Table 1 (four sister cultures). Representative images are displayed. Scale bars = 50 μm.
(TIFF)

S5 Fig Glutamate receptor agonists increase NRG1 type II release. (A) Following transfection of cells with individual NRG1 expression vectors, neuron-enriched cultures were treated with or without GM6001, AP5 and CNQX prior to challenging with vehicle (Control), glutamate (Glu), or PMA as described above. AP immunoreactivity on the surface of living cells was monitored using anti-AP antibodies and a secondary antibody. Scale bar = 50 μm. (B) HEK293 cells transfected with a pRc/CMV vector (Mock), pRc/CMV-AP expression vector (AP), or NRG1 type II-AP expression vector (type II-AP). The enzyme activities of the AP tag on the cell surface were measured 48 h after transfection. Data represent the mean ± SD (three sister cultures each).
(TIFF)

S6 Fig TAPI-0, an inhibitor of ADAM17, blocks the release of NRG1 type II from cortical neuron-enriched cultures. Neuron-enriched cultures transfected with an NRG1 type II-AP expression vector were pretreated with GM6001 (100 nM, 1 h) or TAPI-0 (1 μM, 1 h). Cultures were challenged with saline, glutamate, or NMDA. The enzyme activities of the AP tag in culture supernatants (A) or on the cell surface (B) was measured. Data represent the mean ± SD (three sister cultures each); *p < 0.05, **p < 0.01 vs. control vehicle.
(TIFF)
