# Supplementary figures and images for: Glutamate-dependent ectodomain shedding of neuregulin-1 type II precursors in rat forebrain neurons
Source: PLoS One. 2017 Mar 28;12(3):e0174780. doi: 10.1371/journal.pone.0174780 (PMC5370147; doi:10.1371/journal.pone.0174780)

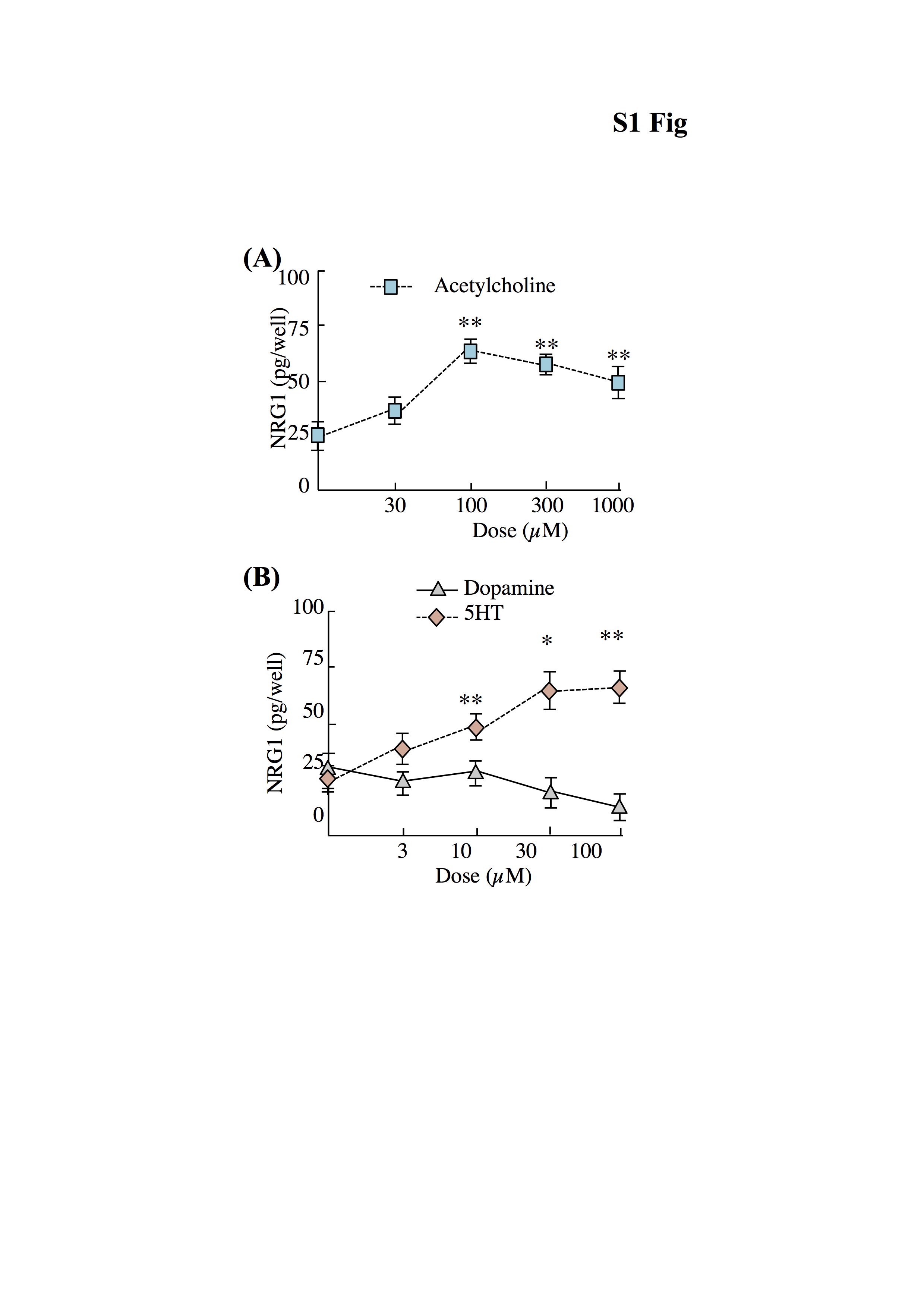

Supplement: S1 Fig — (A) Cultures were treated with 0, 30, 100, 300, 1,000 μM of acetylcholine for 20 min at 37°C. NRG1 concentrations in culture supernatants were measured using ELISA. (B) Cultures were treated with 0, 10, 30, and 100 μM of dopamine or serotonin (5HT). NRG1 concentrations in culture supernatants were measured using ELISA. Data represent the mean ± SD (four sister cultures each); *p < 0.05, **p < 0.01 vs. control vehicle. (TIFF) [file pone.0174780.s001.tiff]

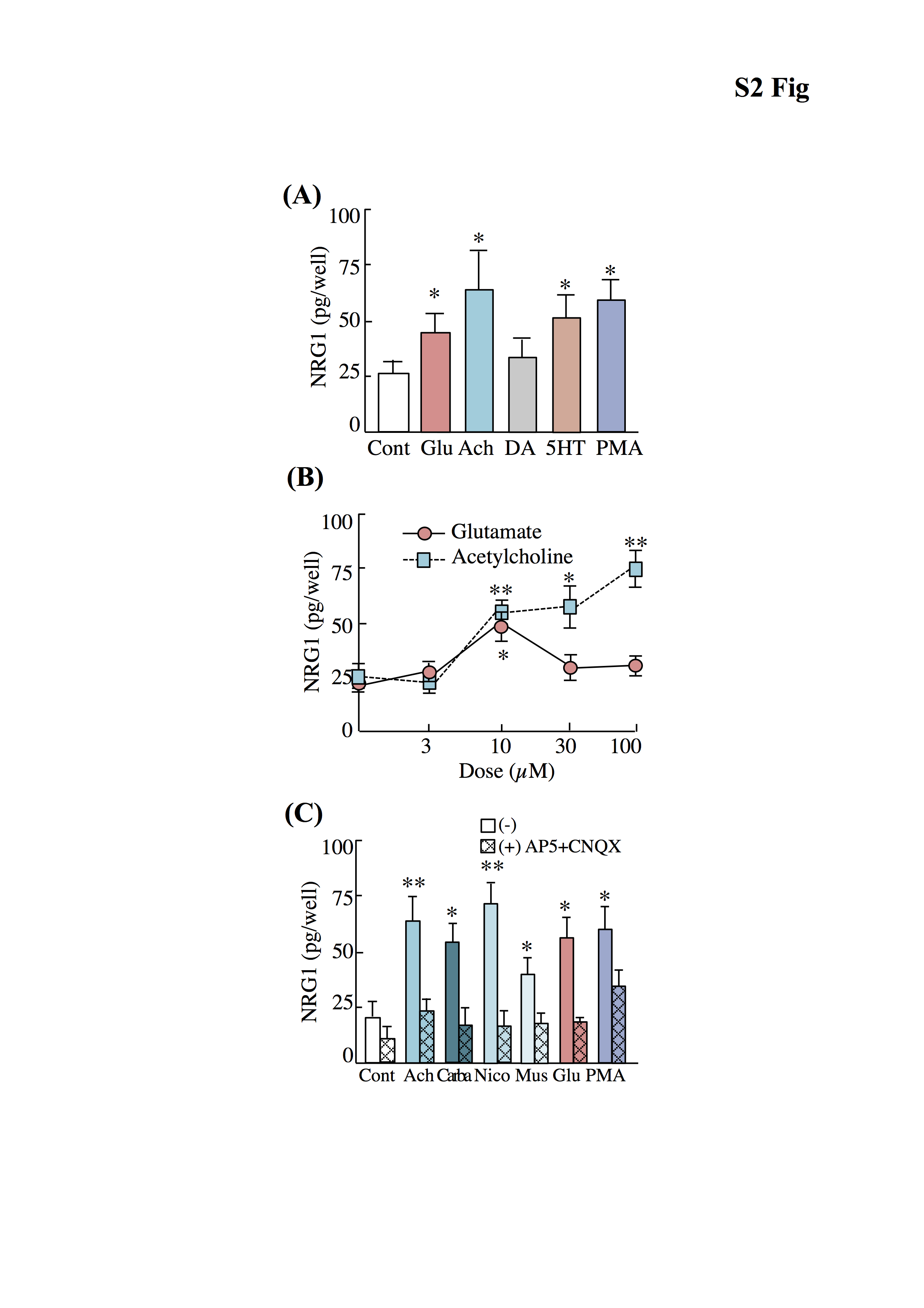

Supplement: S2 Fig — (A) Cultures were treated with control vehicle (Cont), PMA, glutamate (Glu), acetylcholine (Ach), dopamine (DA), or serotonin (5HT) on DIV7. (B) Cultures were treated with 0, 10, 30, and 100 μM of glutamate or acetylcholine. (C) Effects of glutamate receptor antagonists and acetylcholine receptor agonists on NRG1 release. On DIV7, cultures were pretreated with AP5 and CNQX or left untreated, and then challenged with control vehicle (Cont), acetylcholine (Ach), carbachole (Carba), nicotine (Nico), glutamate (Glu), or PMA. NRG1 concentrations in culture supernatants were measured using ELISA. Data represent the mean ± SD (four sister cultures each); *p < 0.05, **p < 0.01 vs. control vehicle. (TIFF) [file pone.0174780.s002.tiff]

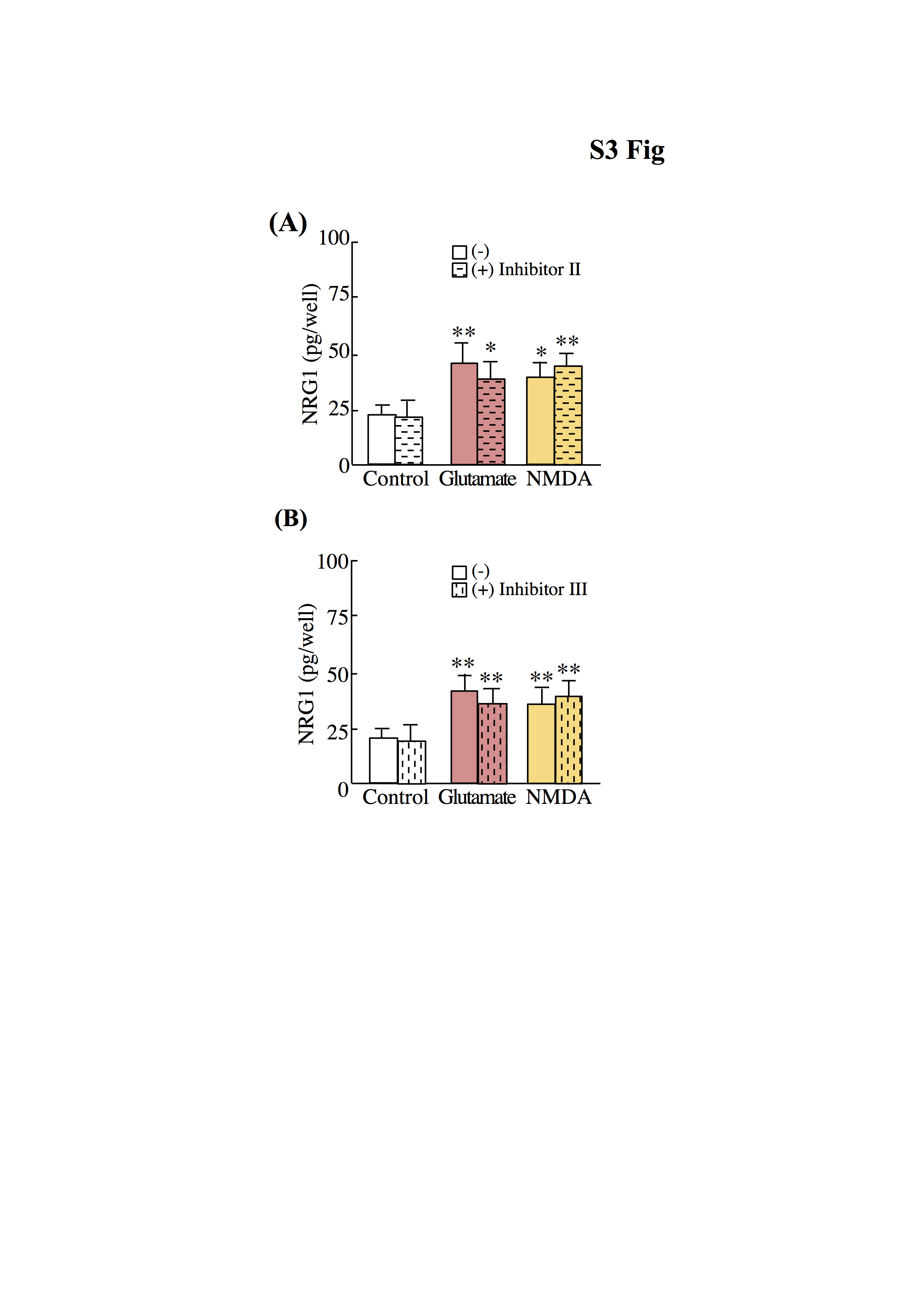

Supplement: S3 Fig — (A) On DIV7, cultures were pretreated with BACE inhibitor II (50 μM, 2 h) and then challenged with control vehicle (Cont), glutamate (10 μM, 20 min), or NMDA (100 μM, 20 min). NRG1 concentrations in culture supernatants were measured using ELISA. (B) On DIV7, cultures were pretreated with BACE inhibitor III (10 μM, 2 h) and then challenged with control vehicle (Control), glutamate (10 μM, 20 min), or NMDA (100 μM, 20 min). NRG1 concentrations in culture supernatants were measured using ELISA. Data represent the mean ± SD (four sister cultures each); *p < 0.05, **p < 0.01 vs. control vehicle. (TIFF) [file pone.0174780.s003.tiff]

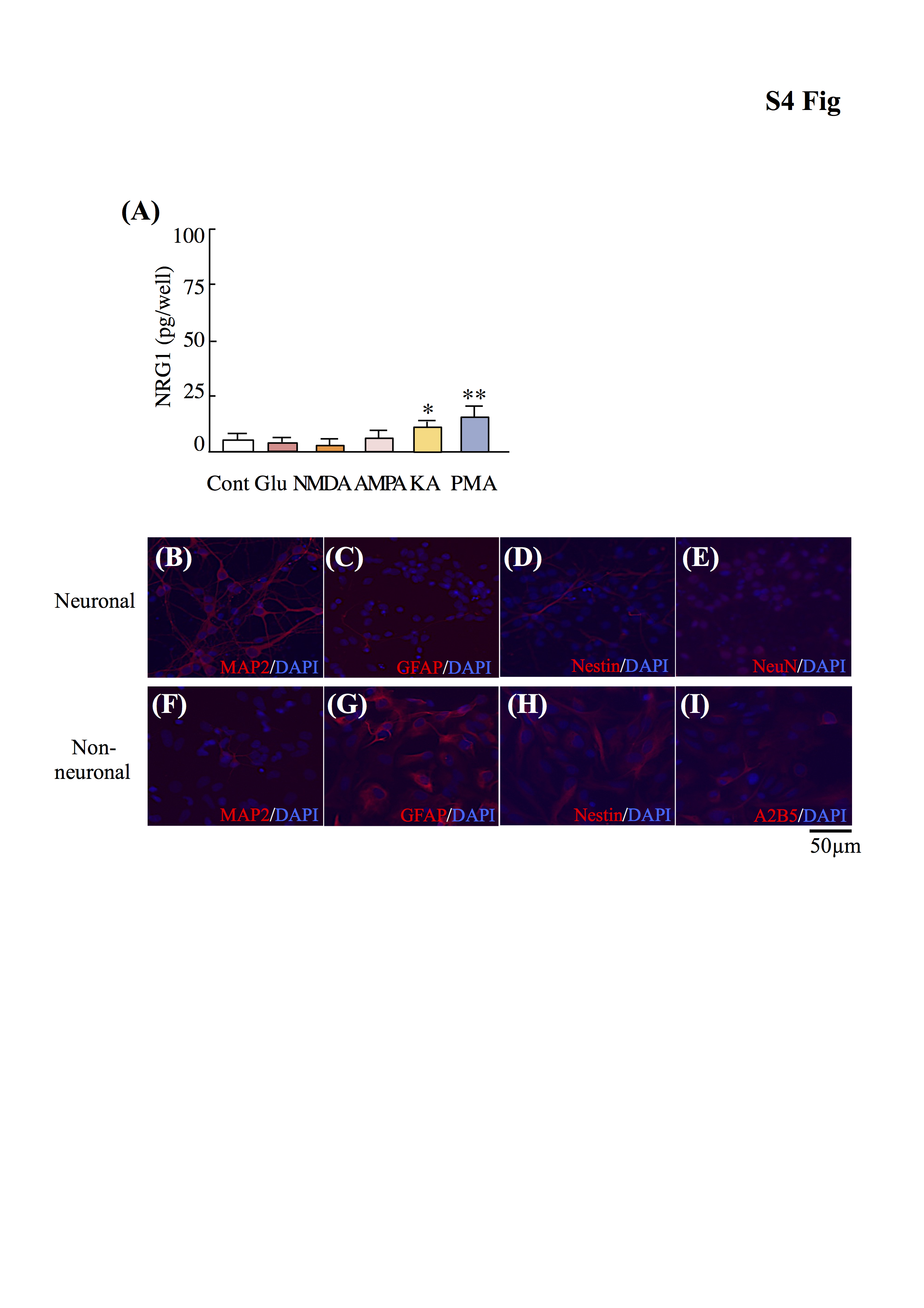

Supplement: S4 Fig — (A) Glia-enriched cultures were challenged with control vehicle (Cont), glutamate (Glu, 10 μM, 20 min), NMDA, AMPA, KA (100 μM, 20 min), or PMA (1 μM, 30 min). NRG1 concentrations in culture supernatants were measured using ELISA. Data represent the mean ± SD (four sister cultures each); *p < 0.05, **p < 0.01 vs. control vehicle. (B–I) Characterization of neuron-enriched and glia-enriched cultures prepared from embryonic cortical tissues. Neuron-enriched cultures were immunostained with the following antibodies: anti-MAP2 (B), anti-GFAP (C), anti-nestin (D), and anti-NeuN (E), followed by anti-mouse or anti-rabbit immunoglobulin fluorescent secondary antibodies. Glia-enriched cultures were immunostained with the following antibodies: anti-MAP2 (F), anti-GFAP (G), anti-nestin (H), and anti-A2B5 (I), followed by anti-mouse or anti-rabbit immunoglobulin fluorescent secondary antibodies. The frequency of individual cell types is shown in Table 1 (four sister cultures). Representative images are displayed. Scale bars = 50 μm. (TIFF) [file pone.0174780.s004.tiff]

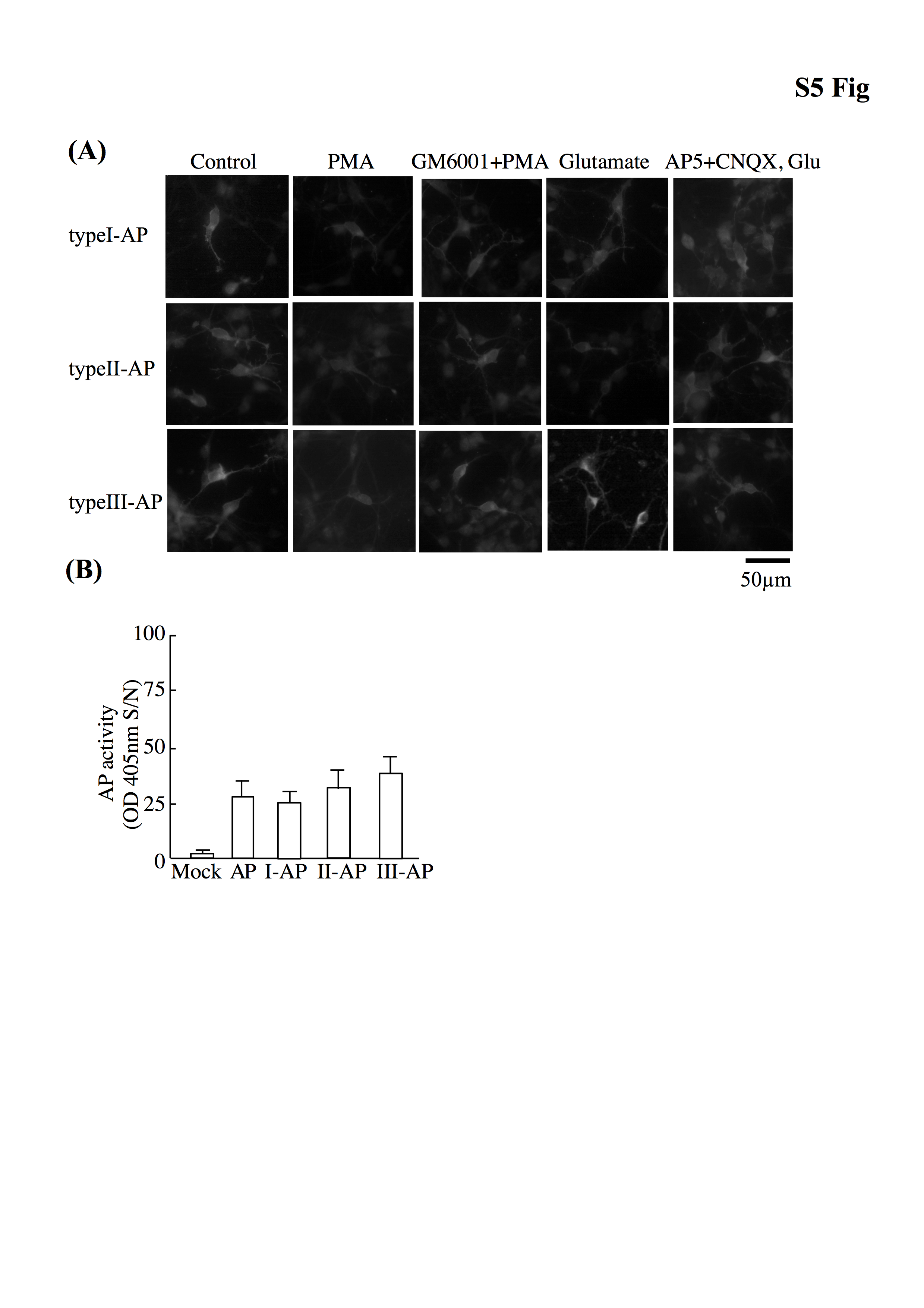

Supplement: S5 Fig — (A) Following transfection of cells with individual NRG1 expression vectors, neuron-enriched cultures were treated with or without GM6001, AP5 and CNQX prior to challenging with vehicle (Control), glutamate (Glu), or PMA as described above. AP immunoreactivity on the surface of living cells was monitored using anti-AP antibodies and a secondary antibody. Scale bar = 50 μm. (B) HEK293 cells transfected with a pRc/CMV vector (Mock), pRc/CMV-AP expression vector (AP), or NRG1 type II-AP expression vector (type II-AP). The enzyme activities of the AP tag on the cell surface were measured 48 h after transfection. Data represent the mean ± SD (three sister cultures each). (TIFF) [file pone.0174780.s005.tiff]

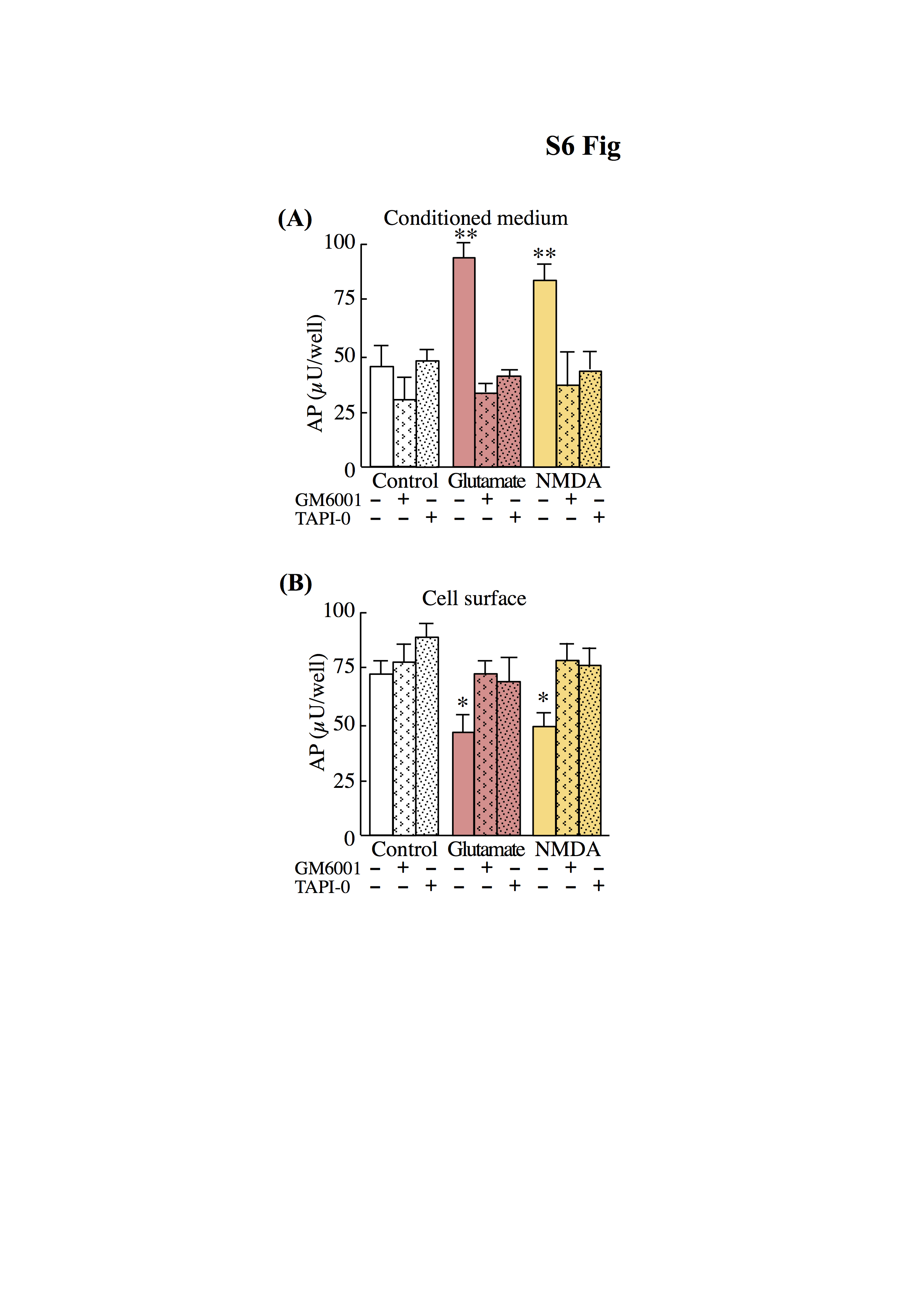

Supplement: S6 Fig — Neuron-enriched cultures transfected with an NRG1 type II-AP expression vector were pretreated with GM6001 (100 nM, 1 h) or TAPI-0 (1 μM, 1 h). Cultures were challenged with saline, glutamate, or NMDA. The enzyme activities of the AP tag in culture supernatants (A) or on the cell surface (B) was measured. Data represent the mean ± SD (three sister cultures each); *p < 0.05, **p < 0.01 vs. control vehicle. (TIFF) [file pone.0174780.s006.tiff]
